# Structuring integration for patient-centered care: a review-informed ontology-driven modular front-end framework for digital health innovation

**DOI:** 10.3389/fdgth.2026.1688261

**Published:** 2026-03-19

**Authors:** Radha Ambalavanan, R Sterling Snead, Julia Marczika, Gideon Towett, Alex Malioukis, Mercy Mbogori-Kairichi

**Affiliations:** Research Department, The Self Research Institute, Broken Arrow, OK, United States

**Keywords:** conceptual framework, data integration, EHR usability, front-end framework, HL7 FHIR, medical ontology, patient-centered care, semantic interoperability

## Abstract

**Background:**

Semantic interoperability remains a significant barrier in healthcare, particularly when integrating patient-reported, clinical, and genomic data to enable personalized care. Existing models rarely focus on patient-centered, ontology-driven front-end architectures based on widely adopted standardized medical ontologies and terminologies. Within broader Personal Health Data Space (PHDS) initiatives, such integration increasingly depends on front-end frameworks that enable semantic consistency and patient-centered usability across heterogeneous clinical domains and systems.

**Objective:**

This analysis presents a review-informed framework to support semantic integration, data governance, user experience, and patient engagement. The objective is to present a front-end, standards-aligned, ontology-driven model grounded in established healthcare standards.

**Methods:**

Based on our previously published systematic review and thematic synthesis, this paper presents a review-informed conceptual framework. It outlines a modular front-end architecture for semantic healthcare data integration. The framework was developed through a reproducible synthesis-to-design process, consistent with design science principles of treatment design, thereby ensuring conceptual rigor and alignment with evidence. Using a knowledge-based modeling approach, we designed a six-layer architecture comprising User Experience, Security and Compliance, Data Management, Interoperability and Integration, Advanced Analytics, and Support and Scalability. Each layer is aligned with established standards including Health Level Seven—Fast Healthcare Interoperability Resources (HL7 FHIR), Systematized Nomenclature of Medicine—Clinical Terms (SNOMED CT), and Logical Observation Identifiers Names and Codes (LOINC), compliance with privacy and security regulations such as the General Data Protection Regulation (GDPR) and the Health Insurance Portability and Accountability Act (HIPAA).

**Results:**

The framework illustrates how ontologies and health IT standards can be conceptually incorporated within front-end system design to unify structured and unstructured data, providing a foundation for secure sharing and standards-aligned integration with existing health information systems.

**Conclusions:**

This review-informed analysis introduces the Self Data Atlas Front-End Framework (SDA-FEF), an ontology-driven, standards-aligned Electronic Health Record (EHR) front-end architecture designed to support patient-centered care. By promoting semantic interoperability, structured data integration, and user-centered design, the framework conceptually advances the development of healthcare systems that may enhance continuity of care and overall quality of life.

## 1 Introduction

With healthcare systems rapidly shifting toward artificial intelligence (AI)-enabled, data-driven care, the urgency to harmonize diverse health data for personalized, patient-centered care has never been greater (1, 2). Globally, achieving this integration is critical to improving care quality, patient safety, and health system efficiency, while also ensuring semantic interoperability and user-friendly front-end design for patient-centered systems.

This conceptual framework also aligns with broader international initiatives, such as the WHO's Global Strategy on Digital Health 2020–2025, which emphasizes interoperability, patient-centered care, and ethical governance in digital health ecosystems (3). Over the past six decades, EHRs have evolved from isolated institutional systems into interconnected platforms. Key milestones include U.S. standardization efforts in 2004, the 2009 HITECH Act, and the incorporation of AI and machine learning to enhance analytics, predictive modeling, and personalized healthcare delivery (4, 5).

While EHRs have improved access to clinical information, they often fall short in enabling seamless data exchange across systems and in supporting structured inputs from patients, caregivers, and non-traditional health sources (6–8). However, there is currently no widely adopted front-end framework that combines semantic interoperability with patient-centered usability. A persistent gap is semantic interoperability, which is the ability of systems to not only exchange data, but also interpret and apply it consistently across contexts (9).

Ontologies offer a promising solution to this challenge by providing standardized vocabularies and structured relationships between medical concepts (10). Frameworks such as Health Level Seven—Fast Healthcare Interoperability Resources (HL7 FHIR), Systematized Nomenclature of Medicine—Clinical Terms (SNOMED CT), and Logical Observation Identifiers Names and Codes (LOINC) have helped standardize clinical data. However, integrating these into usable, patient-centered systems remains complex and often lacks modularity and real-world usability (11, 12). In particular, patient-centered applications often lack the semantic depth and modular architecture needed to handle phenotypic, genotypic, and behavioral data while preserving usability and compliance with privacy regulations (13, 14).

Although several studies have explored ontology-based systems and data models (15), few have focused on translating these models into front-end design concepts. Most existing models focus either on back-end interoperability or technical ontological representations, with minimal attention to real-world usability and modularity in patient-centered applications. The Self Data Atlas Front-End Framework (SDA-FEF) addresses these limitations by combining semantic interoperability with a modular, user-centered front-end architecture, providing greater adaptability and standards alignment than existing frameworks.

To address this, we present a review-informed conceptual framework, the SDA-FEF developed through thematic synthesis from our prior systematic review of 161 studies (16). The framework is a conceptual, standards-aligned, modular, ontology-driven conceptual design, in which data elements and relationships are structured using standardized medical ontologies to support consistent interpretation across systems, addressing usability and semantic interoperability gaps in front-end Electronic Health Records (EHR) systems for patient-centered digital health integration. The resulting six-layer architecture integrates user experience design, compliance mechanisms, data governance, and semantic standards to inform patient-centered, standards-based interoperability. The objective of this paper is to present the design and theoretical underpinnings of SDA-FEF, which bridges semantic architectures with practical, patient-centered usability in front-end EHR design, supports the integration of patient-reported, clinical, and genomic data within a unified framework, and align with internationally recognized standards. Through this approach, it serves as a pathway for future implementation and evaluation, offering a scalable, modular, and standards-aligned approach toward personalized, interoperable digital healthcare. To illustrate its practical relevance and design rationale, the following subsections present motivating use cases in chronic disease management. Chronic conditions such as Long COVID and Type 2 Diabetes demonstrate the fragmentation of digital health data across systems, where laboratory results, clinical notes, and patient inputs often remain disconnected. The proposed conceptual framework (SDA-FEF) addresses this gap by linking diverse information streams through standardized medical terminologies, supporting a unified and meaningful interpretation of information for both clinicians and patients.

### 1.1 Motivating use cases: chronic disease management in long COVID and type 2 diabetes

Chronic conditions such as Long COVID, diabetes, hypertension, cardiovascular disease, and autoimmune disorders share similar challenges in digital health management, including fragmented data architectures, limited interoperability across EHR systems, and inconsistent alignment with clinical guidelines (3). In simple terms, information that should flow seamlessly between clinicians and patients is often locked in separate systems. Effective management of such conditions requires longitudinal, integrated frameworks that link patient-reported outcomes, laboratory measures, and treatment plans to decision-support tools within the clinical workflow (17). To demonstrate these challenges and opportunities, SDA-FEF is contextualized through two representative use cases: Long COVID, which highlights the need to connect diverse symptom and diagnostic data across domains, and Type 2 Diabetes, which illustrates how structured, guideline-based care could be digitally supported in primary practice.

#### 1.1.1 General interoperability gap and stakeholder model

At present, clinical data remain scattered across disparate EHR modules and external systems. Clinicians must navigate multiple screens and coding standards, while patients often have an incomplete understanding of their goals and care actions (18). This conceptual gap underscores the rationale for developing the SDA-FEF, a modular, ontology-driven front-end framework that integrates standardized FHIR resources and semantic terminologies such as SNOMED CT, LOINC, and RxNorm to create a unified, patient-centered interface supporting guideline-aware care delivery at the point of care.

Stakeholders include patients, general practitioners, nurses, and data-service teams who interact with information at different points of care. SDA-FEF organizes standardized FHIR resources such as Patient, Encounter, Observation, Condition, Medication Request, Care Plan, Immunization, and Questionnaire Response, alongside standardized semantic terminologies, such as SNOMED CT for clinical concepts, LOINC for laboratory measures, and ATC or RxNorm for medications. Together, these elements create a common “language” between systems and users, allowing clinical information to remain interpretable and consistent across systems and contexts.

#### 1.1.2 Type 2 diabetes: a structured chronic-care model

Type 2 diabetes serves as a well-established model for evidence-based and guideline-driven chronic-care management. As outlined in the NICE guidelines (NG28) (19) and the American Diabetes Association's 2024 Standards of Care (20), annual reviews require reconciling longitudinal data such as glycated hemoglobin (HbA1c), estimated glomerular filtration rate (eGFR), lipid profile, blood pressure, and body-mass index, along with complication screening and lifestyle assessment. However, these data often remain fragmented across EHR systems, requiring manual navigation and code reconciliation. The SDA-FEF conceptually supports a single, unified front-end view (for example, a dashboard) that could aggregate longitudinal clinical indicators and enable comparison against established guideline targets, conceptually facilitating structured clinical review and patient understanding.

#### 1.1.3 Long COVID: an emerging model for post-viral chronic management

A similar integration challenge exists in Long COVID, where heterogeneous and evolving symptoms and variable care pathways complicate identification and long-term follow-up. Our previous study conceptualized an ontology-based representation of Long COVID symptom trajectories using the Human Phenotype Ontology (HPO) and HL7 FHIR integration (21). Complementary large-scale analyses from the N3C consortium demonstrated that EHR-based machine learning can identify probable Long COVID cases and inform clinical triage for specialized care (17). The SDA-FEF conceptually supports a single, unified front-end view (for example, a dashboard) that could aggregate heterogeneous and evolving symptom data, supporting pattern recognition and longitudinal review across care pathways, thereby facilitating clinical interpretation and patient understanding. Beyond diabetes and Long COVID, the framework is scalable to other chronic conditions, including hypertension, rheumatoid arthritis, and neurodevelopmental disorders, which present similar challenges of longitudinal data integration and cross-system interoperability.

### 1.2 Framework evolution and integration with emerging digital health models

The SDA-FEF framework builds on our earlier work on chronic condition management and digital health informatics integration (21). This foundation enabled the representation of chronic conditions within a unified semantic structure, which SDA-FEF is intended to extend conceptually to broader primary care and long-term disease management. In practical terms, the same digital logic that once tracked Long COVID symptoms could be applied to chronic diseases like diabetes or hypertension, making the framework highly reusable. Recent developments, such as the Patient Medical Digital Twin (PMDT) model (22), further demonstrated the use of ontology-driven architectures to integrate clinical, behavioral, and genomic data for precision medicine. These advances illustrate a broader trend in healthcare, moving from data collection toward data understanding through semantic intelligence, the core principle behind SDA-FEF.

### 1.3 Policy and interoperability alignment

The SDA-FEF framework's emphasis on semantic interoperability, data security, and standardized patient summaries aligns with international eHealth recommendations for cross-border data exchange, including the EU4Digital Common Guidelines for eHealth Harmonization and Interoperability (23). This alignment supports the framework's conceptual suitability for cross-border data exchange and governance. By translating these principles into a modular ontology-driven conceptual design, SDA-FEF provides a scalable foundation for future implementation of trustworthy, standards aligned chronic care solutions across health systems. Future work will be needed to evaluate how these design principles perform in operational settings and how they influence patient- and clinician-centered experiences.

## 2 Theoretical foundations

### 2.1 Literature and theoretical background

The development of health information systems capable of supporting personalized and patient-centered care requires not only technological integration; but also, a robust conceptual foundation that unifies diverse data sources in a semantically coherent and practically usable structure (24, 25).

Conceptual frameworks play a pivotal role in organizing health system components around specific goals (26, 27). In the context of semantic interoperability, a conceptual framework must do more than define data flows; it should embed meaningful relationships, contextual cues, and regulatory boundaries within the system architecture (28).

The framework's structure is aligned with HL7 FHIR, SNOMED CT, LOINC, the Health Insurance Portability and Accountability Act (HIPAA), and the General Data Protection Regulation (GDPR) (11, 29). These standards not only provide semantic consistency but also align with regulatory requirements, facilitating cross-platform compatibility and trust among users. Furthermore, the integration of phenotypic and genotypic data into patient profiles reflects a growing emphasis on precision medicine and personalized care delivery, supported by ontology-driven reasoning (30–32).

### 2.2 Baseline workflow and gap analysis

To situate the proposed framework within current clinical informatics practice, this subsection reviews existing EHR workflows, outlines their limitations, and defines the interoperability and usability gaps that SDA-FEF seeks to address. In present EHR systems for chronic disease management, clinicians often navigate multiple unlinked modules to review longitudinal indicators such as HbA1c, lipid profiles, blood pressure, or renal function results, reconcile medication lists and contraindications, and verify completion of required follow-up or screening activities (18, 33). Documentation of care goals and outcomes is frequently captured in free text or static templates, detached from computable care-plan structures (structured digital care plans that systems are intended to read and track). These constraints stem from interfaces organized by data source rather than clinical task, heterogeneous coding systems that reduce semantic consistency, limited alignment between guideline logic and user-interface elements, and minimal integration of patient-reported or self-tracking data (34).

The SDA-FEF is proposed as a conceptual response to these deficiencies by integrating semantic interoperability with task-oriented usability. It outlines how dynamic composition of data from multiple sources, semantic normalization across clinical terminologies, and guideline-aware interactions could be supported through ontology-aligned front-end design. The framework also conceptualizes bi-directional, computable care-plan representations as a future capability to support continuity of care across chronic conditions. This direction is consistent with global interoperability initiatives, including the LOINC–SNOMED CT Ontology 2.0 collaboration, which strengthens semantic alignment for laboratory and clinical data exchange (35).

### 2.3 Proposed solution and use-case improvements

Building on this conceptual foundation, the SDA-FEF is presented as a structured solution to the fragmentation and limited task alignment identified in current EHR workflows. It defines a modular, ontology-driven front-end architecture that integrates standardized FHIR resources with domain ontologies to support patient-centered and guideline-aware interface design. Within chronic-care contexts, this conceptual framework illustrates how longitudinal indicators such as HbA1c, eGFR, lipid profiles, blood pressure, and care-plan adherence could be organized and presented within a unified front-end view to support both clinician review and patient understanding (18).

At a conceptual level, the ontology layer harmonizes terminologies from SNOMED CT, LOINC, and ATC/RxNorm, ensuring semantic equivalence across data sources and modules (35). Guideline knowledge (for example, NICE NG28 and the ADA Standards of Care) is treated as an external reference that can inform how data are structured and contextualized at the front-end interface, without implying real-time execution or automated decision-making. From a usability perspective, the framework conceptualizes bi-directional, computable care-plan representations as a future capability, illustrating how patient-reported outcomes and lifestyle updates could be aligned with clinician-facing views to support continuity of care. Collectively, this section demonstrates how SDA-FEF reframes fragmented EHR interactions into a conceptually interoperable, task-oriented, and patient-centered workflow, aligned with the World Health Organization's digital health interoperability priorities (3).

### 2.4 Next steps and complex aspects

The next stage of SDA-FEF focuses on extending the front-end framework toward real-world configurability while maintaining its current conceptual scope. Future refinements will address three areas: (i) enhancing semantic alignment across heterogeneous systems through SHACL-based validation and terminology governance using LOINC and SNOMED CT (36); (ii) planning future evaluation of interoperability through bi-directional data exchange guided by HL7 FHIR profiles (36); and (iii) planning usability assessments drawing on established health-IT methodologies (37). Given the Hypothesis and Theory format, low-level API or data-flow diagrams are not included, but the manuscript now clearly defines front-end system boundaries and expected interactions with standards-based back-end systems. Together, these directions outline a practical pathway for SDA-FEF's evolution, ensuring scalability, governance, and patient-centered interoperability in alignment with global frameworks such as the WHO Digital Health Strategy (2020–2025) (3) and EU4Digital (23). In future stages, pilot implementations using non-clinical synthetic datasets will be explored to assess interoperability and usability before progressing toward real-world clinical validation. These evaluations will remain within the framework's conceptual trajectory, serving as a bridge between theory-driven design and applied digital health deployment.

### 2.5 Design mapping rationale

The design decisions are grounded in systematic review evidence to ensure alignment with established research findings. However, operationalizing ontologies within patient-centered applications remains a persistent challenge in digital health innovation (38, 39).

A high-level, review-informed mapping of the identified themes to corresponding framework layers is provided to illustrate how evidence informed each functional component. SDA-FEF builds on this understanding by organizing front-end components into distinct but interconnected conceptual layers, each responsible for a core function including patient engagement, privacy and compliance, data harmonization, and advanced analytics.

In synthesizing these theoretical foundations, this framework serves as a bridge between semantic models and the real-world demands of usability, governance, and patient-driven healthcare. Table 1 presents a review-informed, conceptual mapping that illustrates how the core themes identified through our previously published systematic review and thematic synthesis informed the design of the SDA-FEF layers, providing clear traceability between prior evidence and the current framework structure. Detailed supporting materials are provided in Supplementary File S1 as a multi-sheet Excel workbook, including the articles database compiled from multiple sources (Supplementary Sheet 1), curated article list (Supplementary Sheet 2), categorized thematic summaries (Supplementary Sheet 3), annotation details for each included article (Supplementary Sheet 4), and extended evidence-to-framework mapping and methodological alignment materials (Supplementary Sheet 5).

**Table 1:** Review-informed mapping of evidence-derived themes to conceptual SDA-FEF architectural layers.

Review-derived theme (from prior systematic review and thematic synthesis)	Key evidence identified in the literature	Corresponding SDA-FEF conceptual layer	Front-end role supported
Semantic interoperability gaps across EHR systems	Fragmented clinical data representations, lack of shared semantic meaning, inconsistent reuse of standardized terminologies (HL7 FHIR, SNOMED CT, LOINC)	Interoperability and Integration Layer	Ensures consistent interpretation and presentation of clinical concepts across patient-centered and clinician-centered interfaces through ontology-aligned data representations
Limited patient-centered data integration and engagement	Poor integration of patient-reported outcomes, preferences, and contextual data into digital health interfaces, limiting engagement and shared decision-making	User Experience Layer	Enables patient-friendly visualization, navigation, and interaction with health data, supporting engagement and comprehension at the front-end
Challenges in integrating multi-source health data	Difficulties combining EHR data with patient-generated, wearable, and genomic data in a coherent and usable manner	Data Management Layer	Supports unified front-end views of heterogeneous data sources while abstracting underlying technical complexity from end users
Trust, transparency, and data governance concerns	Need for transparency, traceability, consent awareness, and trust as prerequisites for patient engagement and long-term adoption of digital health systems	Security and Compliance Layer	Communicates data provenance, consent status, and governance cues to end users, supporting trust and regulatory compliance
Need for advanced interpretation and analytic support	Growing demand for meaningful interpretation of complex, integrated clinical data to support decision-making	Advanced Analytics Layer	Supports front-end delivery of analytics-driven insights and summaries while maintaining separation from back-end analytic engines
Scalability and system evolution challenges	Need for systems to scale across populations, data volumes, and evolving use cases without compromising usability	Support and Scalability Layer	Enables front-end adaptability and performance across expanding datasets, user groups, and functional requirements

In this work, the term ontology-driven refers to the use of ontology-aligned data representations to guide how health information is organized, labeled, and presented at the front-end interface, rather than to real-time reasoning or inference performed within the user interface itself. As an illustrative example, when a patient views blood pressure data in the front-end interface, ontology alignment enables systolic and diastolic values to be treated as components of a single clinical concept rather than as isolated measurements. This supports consistent grouping of related values, standardized terminology, and context-aware presentation aligned with clinical meaning. This example is illustrative and intended to demonstrate front-end structuring rather than system behavior.

The same ontology-aligned structure supports clinician-facing views by enabling consistent labeling, comparison across encounters, and linkage to related clinical concepts such as conditions or medications. Importantly, terminology services, reasoning, and rule execution remain external to the front-end. Accordingly, ontology-driven design informs what is presented and how it is structured at the user interface level, without implying implementation-level analytics or back-end decision logic within the front-end itself.

To clearly delineate system boundaries, this section clarifies the scope of the proposed front-end framework. The framework is positioned as a conceptual front-end architecture that governs how health information is structured, labeled, and presented to end users. Functions such as terminology matching, semantic reasoning, rule execution, and decision logic are not performed within the front-end itself. Instead, these capabilities are provided by external terminology and analytics services or other back-end components, whose outputs inform front-end organization and presentation through ontology-aligned data representations. In this way, the framework focuses on front-end responsibilities related to usability, consistency, and interpretability, without implying implementation of back-end analytics or reasoning mechanisms within the user interface layer.

While layered architectures are common in health information systems, the contribution of this work lies in how the proposed framework is derived and scoped. Unlike generic architectural models, the SDA-FEF framework is clearly informed by evidence synthesized from a prior systematic review and thematic analysis, with each front-end layer mapped to recurrent challenges identified in the literature. The framework is intentionally focused on the front-end domain, which is often under-specified in existing health IT architectures, and articulates how ontology-aligned data representations can support consistent, interpretable, and patient-centered presentation of health information. By providing clear semantic traceability between review-derived themes, conceptual layers, and front-end roles, the framework offers a review-informed design rationale rather than a purely conceptual or implementation-driven architecture. Accordingly, all results, use cases, and architectural descriptions in this study are intended as conceptual illustrations rather than reports of implemented or empirically tested systems.

## 3 Methods: framework development through conceptual design and thematic synthesis

Thematic synthesis from a registered systematic review in the Open Science Framework (OSF) informed the framework (40). No empirical data were collected. The detailed mapping process, briefly summarized in Theoretical Foundations, is expanded here to describe how the framework was designed. The evidence synthesis identified critical design requirements related to semantic interoperability, patient-centered care, genomic data integration, and secure data sharing infrastructures.

Categories from the review were mapped to six functional layers, each addressing a specific combination of usability, governance, and semantic needs. This process involved translating thematic synthesis findings into domain-specific conceptual entities and relationships, guided by semantic interoperability standards and ontology design principles. An iterative modeling approach, supported by structured synthesis techniques such as concept mapping, was used to construct the visual and functional organization of the framework. Components were conceptually mapped to HL7 FHIR, SNOMED CT, and LOINC to ensure consistency.

### 3.1 Framework development process

The methodological approach was guided by Design Science Research Methodology (DSRM), following Wieringa's framework, and applied conceptually to support the synthesis and structuring of the proposed framework while maintaining its theoretical nature (41). The SDA-FEF development process was structured into three iterative stages: evidence synthesis, thematic mapping, and layered architecture design, providing a clear methodological foundation for the framework.

1. **Evidence synthesis**: Drawing on thematic synthesis from qualitative and observational studies to identify key principles, including user-centered design, regulatory compliance, modular architecture, and standardized terminology integration.
2. **Thematic mapping**: Aligning these principles with conceptual domains such as user experience, security and compliance, data management, interoperability, analytics, and scalability.
3. **Layered architecture design**: Arranging these domains across six functional layers, ensuring each is modular, evidence-driven, and aligned with interoperability standards and global privacy and security regulations.

#### 3.1.1 Framework derivation and conceptual modeling

The SDA-FEF framework was derived from a registered systematic review (*n* = 161 studies) that synthesized design requirements for digital health data integration. No formal ontology file or data structure was created at this stage. Terminology services, reasoning, and rule execution are handled by external systems and are not performed within the front-end. The front-end is limited to reflecting their outputs through ontology-aligned structure and presentation. Instead, thematic categories from the review were translated into domain entities and relationships aligned with HL7 FHIR resources (Patient, Observation, Condition, Medication Request, Care Plan, Immunization, Questionnaire Response) mapped conceptually to international terminologies, including SNOMED CT, LOINC, RxNorm/ATC, and HPO. These were organized into six functional layers: (i) User Experience; (ii) Security and Compliance; (iii) Data Management; (iv) Interoperability and Integration (v) Advanced Analytics; and (vi) Support and Scalability, to ensure modular representation of usability, governance, and semantic needs. The mappings and architecture were reviewed for coherence and completeness through internal expert discussions. Formal ontology development using OWL 2 and SHACL, along with public repository registration, are planned as part of future implementation and evaluation work beyond the present conceptual phase.

### 3.2 Ontology and standards integration

The framework's structure is aligned with HL7 FHIR for data exchange, SNOMED CT and LOINC for clinical terminology, and GDPR and HIPAA for privacy and security compliance. These standards were selected for their established adoption in health informatics and direct alignment with the thematic requirements derived from evidence synthesis.

### 3.3 Diagram construction

Two conceptual diagrams were constructed to illustrate the layered architecture and hierarchical relationships within the front-end framework. These diagrams were generated using concept mapping tools to ensure alignment with the thematic structure and design rationale. Concept mapping was selected for its ability to visually represent complex modular interdependencies, making the relationships between layers and ontology-driven components interpretable for diverse stakeholders.

### 3.4 Treatment validation

Following Wieringa's Design Science Methodology, the proposed SDA-FEF is validated conceptually through justificatory reasoning rather than empirical evaluation. Validation is performed by checking whether SDA-FEF satisfies the derived design requirements (R1–R10; Table 2) through architectural reasoning, standards alignment, and evidence traceability, predicting its expected effects in the intended context. This validation is based on three elements: (i) alignment of the framework's layers and roles with requirements derived from the systematic review and thematic synthesis and stakeholder goals, (ii) conformance with established interoperability and terminology standards (including HL7 FHIR, SNOMED CT, LOINC, and RxNorm), and (iii) architectural reasoning regarding the interaction between front-end structure and its intended healthcare context. On this basis, the framework is expected to support improved semantic consistency, reduced information fragmentation, clearer patient-facing representations that support patient-centered care, and stronger governance and compliance at the front-end. This validation remains conceptual and predictive in nature. Empirical validation and implementation-level evaluation will be addressed in future work. A detailed audit of the study's alignment with Wieringa's Design Science Methodology is provided in Supplementary File S1 (Sheet 5).

**Table 2:** Derived design requirements for SDA-FEF.

Requirement ID	Design requirement	Evidence source
R1	The front-end framework shall support semantic interoperability across heterogeneous EHR systems by aligning clinical data representations with established standards (e.g., HL7 FHIR, SNOMED CT, LOINC).	Systematic review findings on semantic interoperability gaps
R2	The framework shall enable consistent interpretation and presentation of clinical concepts across patient-facing and clinician-facing interfaces through ontology-aligned data structures.	Thematic synthesis of interoperability and usability studies
R3	The front-end shall integrate patient-reported outcomes, preferences, and contextual data to support patient-centered engagement and shared decision-making.	Evidence on limited patient-centered data integration
R4	The framework shall support unified front-end views of multi-source health data, including EHR, wearable, and genomic data, while abstracting underlying technical complexity from end users.	Review evidence on challenges in multi-source data integration
R5	The front-end shall provide transparent communication of data provenance, consent status, and governance cues to support user trust and regulatory compliance.	Literature on trust, transparency, and data governance
R6	The framework shall separate front-end presentation logic from back-end data processing and analytics engines to preserve modularity and maintainability.	Architectural principles identified across reviewed studies
R7	The front-end shall support meaningful interpretation and summarization of complex integrated data to facilitate clinical and patient decision-making.	Evidence on the need for advanced interpretation and analytic support
R8	The framework shall be adaptable to evolving clinical use cases, data volumes, and user groups without compromising front-end usability.	Scalability and system evolution challenges reported in the literature
R9	The framework shall align with international digital health strategies and interoperability policies to ensure broader applicability and governance compatibility.	Policy-aligned evidence (WHO, EU4Digital, standards guidance)
R10	The framework shall maintain a clearly defined front-end scope, explicitly excluding back-end implementation and empirical evaluation, to support conceptual validation within a design science methodology.	Methodological constraints and scope definition in the manuscript

Reflects conceptual (justificatory) validation through alignment between review-derived design requirements and the proposed framework. Empirical validation and implementation are explicitly positioned as future work.

## 4 Results: SDA-FEF framework architecture

The SDA-FEF front-end architecture comprises six interrelated layers, each contributing to a unified, semantically aligned digital health experience. This layered approach is intended to support modular development, long-term scalability, and personalization across healthcare contexts. Each layer is evidence-linked to the thematic synthesis findings from the review, ensuring that functional design aligns with identified user, governance, and interoperability needs.

### 4.1 User experience layer

The outermost layer focuses on patient and provider interactions. It emphasizes accessibility, intuitive design, data visualization, and engagement mechanisms tailored for diverse users. Features within this layer include multilingual support, customizable dashboards, and visual indicators of health trends. It is designed to promote active participation in symptom tracking, behavior monitoring, and interaction with intelligent feedback systems. Planned integration with wearable devices and mobile apps is intended to enable continuous and context-aware data collection.

### 4.2 Security and compliance layer

This layer ensures the protection of sensitive health information by implementing robust data security protocols. It supports encryption standards, authentication mechanisms, and role-based access controls. Compliance with international regulations such as GDPR and HIPAA is embedded into the system's architecture. In addition, this layer includes auditing and logging capabilities to maintain accountability and transparency in data handling. These features collectively create a trusted digital environment for both patients and healthcare providers. Design decisions in this layer are informed by evidence from the prior review regarding regulatory compliance and trust-building mechanisms.

### 4.3 Data management layer

The Data Management Layer governs how health data is stored, processed, and maintained. It includes data structuring protocols, metadata tagging, backup mechanisms, and performance monitoring. This layer ensures that structured and unstructured data, such as clinical narratives, self-reported data, and test results, are organized and accessible in a consistent format. It also provides the foundation for data availability and system responsiveness required for high-traffic health applications.

### 4.4 Interoperability and integration layer

At the core of the semantic architecture, this layer is oriented toward standardized data exchange between systems, drawing on HL7 FHIR and ontology-based mappings to SNOMED CT and LOINC to promote consistency in clinical terminology. It is intended to facilitate bi-directional alignment between existing EHR systems and external repositories at a conceptual level. Ontology-based mappings contribute to semantic enrichment of health data, supporting machine interpretability and cross-platform compatibility. Collectively, this layer illustrates how interoperability and integration can be structured to support continuity of care, cross-institutional collaboration, and data reuse within a standards-aligned front-end framework.

### 4.5 Advanced analytics layer

This layer represents the conceptual placement of advanced analytics capabilities within the framework. It illustrates how phenotypic and genotypic data streams could be aligned to support personalized risk assessment, treatment optimization, and outcome tracking in future implementations. The inclusion of analytics concepts reflects thematic evidence emphasizing precision-medicine readiness, without implying implementation of predictive models or real-time decision logic within the present framework.

### 4.6 Support and scalability layer

The innermost layer ensures that the system remains adaptable and resilient. It supports modular plug-ins, load balancing, cloud-based scalability, and continuous integration pipelines. It provides support for future extensions such as back-end semantic integration, ontology querying, and real-time reasoning. By separating foundational infrastructure needs from core functional layers, the architecture ensures long-term sustainability and platform independence.

### 4.7 Knowledge diagram and hierarchy

**Figure 1:**
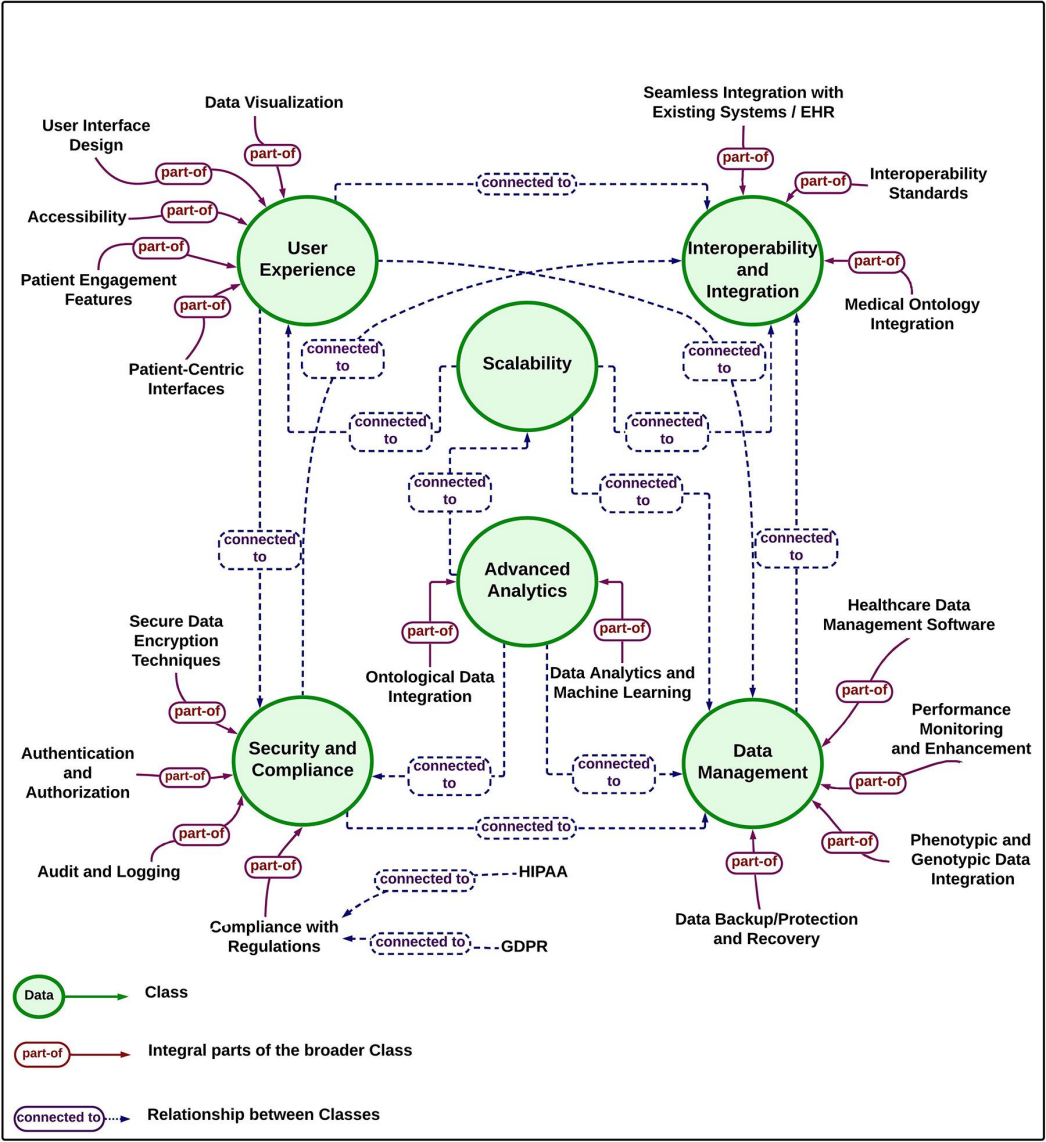
Conceptual overview of the proposed front-end framework. The diagram illustrates the six functional layers of the ontology-driven, patient-centered framework. Arrows indicate conceptual relationships and dependencies between layers, showing how front-end components are informed by semantically harmonized outputs from external services, rather than representing implementation-level data flow, algorithmic execution, or system control logic.

**Figure 2:**
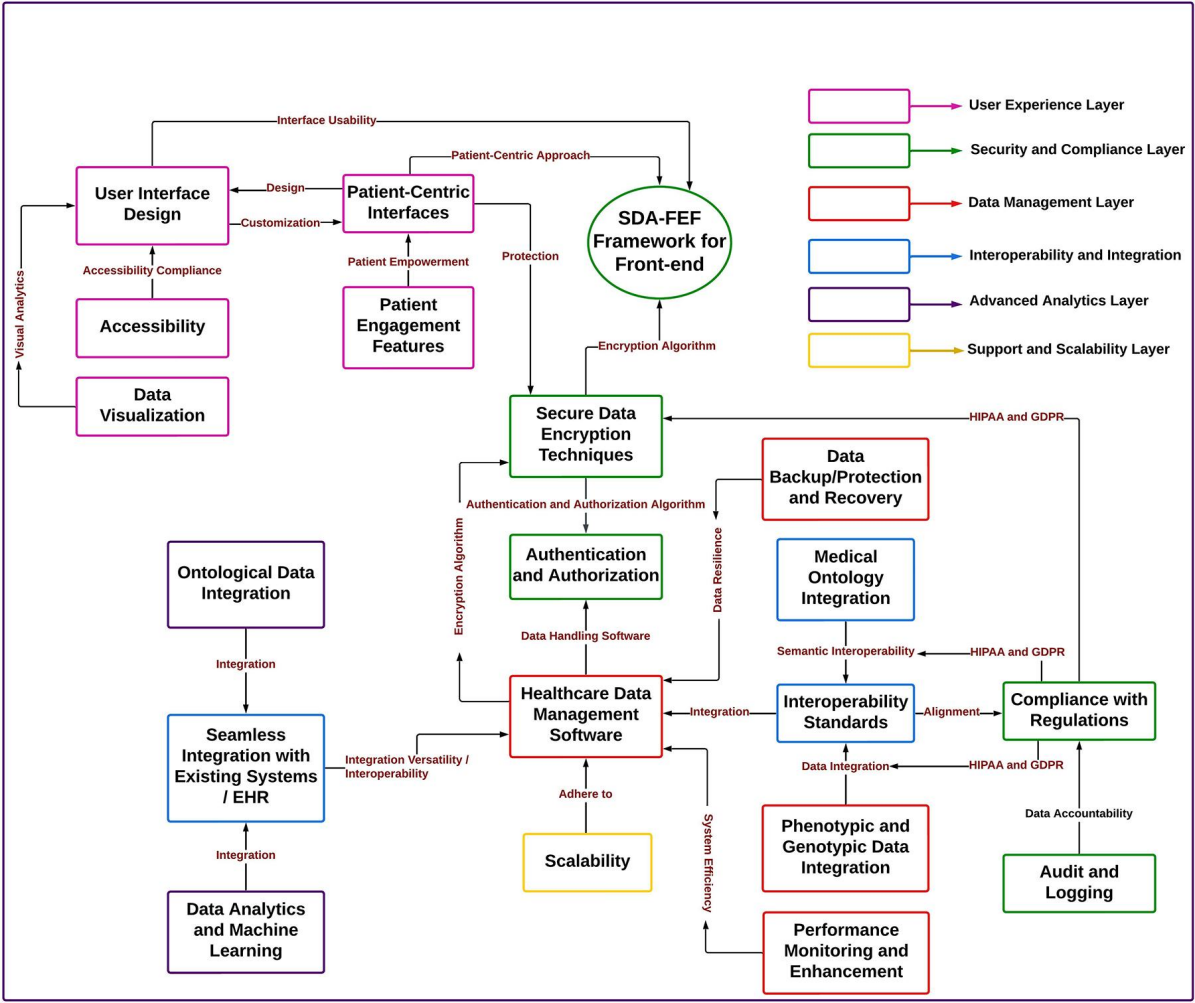
Hierarchical structure and interconnections of components in the SDA-FEF front-end framework. The ontology hierarchy highlights the arrangement of the six functional layers of the SDA-FEF and illustrates the conceptual organization of layers and their interrelationships in supporting scalability considerations, regulatory compliance, and semantic integration. The structure demonstrates the interconnections that support patient-centered usability, modular adaptability, and interoperability with health data standards such as HL7 FHIR, SNOMED CT, and LOINC.

The interconnected SDA-FEF front-end framework layers form an ontology-driven structure (Figure 1) that supports conceptual alignment between patient-centered front-end views and external back-end systems, without executing back-end logic within the front-end layer. The hierarchical relationships among system components are mapped in Figure 2, emphasizing conceptual interconnections, layer responsibilities, and standards alignment. Together, these visualizations illustrate how the six layers function cohesively to support a future-ready architecture for patient-centered digital health systems. These figures are intended to support conceptual understanding of front-end structuring and scope, rather than to depict system behavior or implementation workflows.

To improve interpretability, the figures are designed to emphasize conceptual relationships rather than data or control flow. In the diagrams, arrows indicate conceptual dependencies and relationships between framework layers, illustrating how front-end components are informed by semantically harmonized outputs from external services or shared semantic structures. The arrows do not represent real-time data transfer, algorithmic execution, or system control logic. Instead, they are used to illustrate conceptual interaction and dependency at the architectural level, supporting understanding of layer responsibilities and their interconnections within the proposed front-end framework.

## 5 Discussion

The front end conceptual framework of SDA-FEF provides a structured design foundation for a standards-aligned, modular six-layer architecture that is intended to address the gaps between semantic models and usability. It conceptually responds to limitations identified in current health information systems, including fragmented data collection, semantic interoperability gaps, limited patient engagement, and regulatory compliance challenges (42).

By addressing essential usability challenges in patient-centered applications and persistent semantic interoperability gaps, SDA-FEF outlines a design pathway for translating complex ontological models into user-friendly, standards-aligned digital health solutions. Its modular structure is intended to provide a foundation for targeted development and scalability, while supporting adaptability to evolving precision medicine needs.

### 5.1 Comparisons to existing approaches

Phenotype-driven pipelines such as Exomiser use Human Phenotype Ontology (HPO)-encoded clinical features to prioritize causal variants from whole-exome or whole-genome sequencing. These approaches are widely applied in rare-disease diagnostics, with evidence showing improved diagnostic yield and robustness even when phenotypic data are incomplete or noisy. The 2024 HPO update also documents established mappings between HPO and SNOMED CT, enabling tighter integration with EHR systems and enhancing semantic interoperability. Such mappings support clinical decision support systems (CDSS) that combine patient phenotypic profiles with genomic data for automated reasoning, variant prioritization, and AI-assisted diagnosis. Embedding this type of ontology-driven reasoning within future implementations of the SDA-FEF Advanced Analytics Layer could facilitate high-precision interpretation of multi-modal health data, advancing personalized, standards-aligned care (31, 43–44).

Recent implementations of modular front-end systems in national EHR programs illustrate comparable efforts toward modularity and interoperability. Among these, Ireland's national EHR strategy emphasizes modularity, interoperability, and user-centered design, principles conceptually aligned with SDA-FEF's layered structure (45). Similarly, NHS Digital's IM1 Integration initiative in the United Kingdom demonstrates how modular front-end designs can enable third-party tools to seamlessly interface with core clinical systems in general practice. The IM1 pairing model allows plug-and-play interoperability for patient portals, prescribing systems, and decision support tools, without compromising data protection or system integrity. This mirrors SDA-FEF's scalability and support functions, which accommodate third-party services while preserving core functionality (46). These examples serve as comparative references that demonstrate conceptual alignment with SDA-FEF's design principles and ongoing national interoperability initiatives, rather than empirical validation of the framework.

While these national and institutional initiatives demonstrate that modular architectures and interoperability frameworks can succeed at scale, they often prioritize technical integration with existing infrastructures over semantic depth and patient-centered usability. In contrast, SDA-FEF integrates HL7 FHIR, SNOMED CT, and LOINC within a multi-layered architecture that supports semantic enrichment, regulatory compliance, usability, and scalability from inception. This positions SDA-FEF to bridge the gap between semantic interoperability and real-world usability, a balance that current frameworks do not consistently achieve.

### 5.2 Implications for research and practice

These examples suggest the feasibility and adaptability of modular, patient-centered front-end architectures in national healthcare settings, validating the conceptual direction proposed by SDA-FEF. The integration of international standards such as HL7 FHIR, SNOMED CT, and LOINC ensures that the framework is interoperable across systems and compatible with regulatory requirements. Additionally, the inclusion of GDPR and HIPAA-compliant features demonstrates the framework's attention to ethical and legal obligations, which is essential for building trust among users (47, 48). As noted in the limitations section, this remains a conceptual analysis requiring empirical validation. Future work will focus on empirical implementation and evaluation of the proposed framework in real-world clinical contexts, with a patient-centered design focus.

## 6 Limitations of this conceptual analysis

This conceptual analysis has certain limitations that should be acknowledged. First, the framework remains conceptual and has undergone justificatory validation rather than empirical validation. It has not yet been implemented or evaluated in a clinical or research setting. While the knowledge diagrams and layered architecture offer a structured framework for front-end design, their practical feasibility and user experience have not yet been tested, which may limit the immediate applicability of the model in operational healthcare environments. Consistent with prior work on semantic architectures and standards-based health system design, validation through usability testing and stakeholder feedback will be essential in future work (49, 50).

Second, the current scope focuses on the front-end architecture, with back end semantic components, including ontology servers, API endpoints, and knowledge graphs, discussed conceptually but not yet developed (51, 52). These components will be essential for achieving full semantic functionality in future phases.

The SDA-FEF was informed by evidence from our prior systematic review and supplemented with relevant literature, without undergoing direct empirical testing in this study. This means that while the framework is theoretically grounded and standards-aligned, uncertainty remains about its scalability, interoperability performance, and effectiveness in varied clinical contexts.

## 7 Ethical, legal, and social considerations in genomic data integration

As the SDA-FEF framework advances toward integration of genotypic and phenotypic data, ethical, legal, and social considerations (ELSI) must be thoughtfully addressed to ensure equitable and responsible implementation. Integrating genomic data into healthcare systems raises concerns related to privacy, informed consent, data ownership, and potential misuse, including genetic discrimination and stigmatization (42, 53, 54).

ELSI principles were incorporated at the conceptual design stage to guide alignment with key international data protection regulations, including the GDPR and the HIPAA (55), and to inform adaptive, inclusive consent models that can accommodate the evolving nature of research, data reuse, and clinical care.

Transparency in data governance, secure infrastructures for data sharing, and patient-directed controls over access and usage are critical to maintaining autonomy and promoting active engagement (56–58). These principles are reflected within the proposed framework layers, with the Security and Compliance Layer addressing privacy and data protection considerations, and the User Experience Layer supporting informed consent and engagement.

As genomic technologies become increasingly central to precision medicine, embedding ELSI safeguards within digital health frameworks like SDA-FEF is not optional. Such safeguards are essential to ensuring that technological innovation proceeds hand in hand with ethical responsibility and social trust.

## 8 Conclusions

This study presents the SDA-FEF, a review-informed, ontology-driven conceptual framework developed to address the growing need for structured, secure, and semantically aligned integration of patient-centered health data. By prioritizing usability and modularity, it addresses persistent interoperability and governance challenges, providing a standards-aligned blueprint that bridges semantic models with practical, patient-centered.

Grounded in systematic evidence, the framework establishes a strong conceptual foundation for the design of scalable, interoperable, and policy-aligned front-end health information systems. While clinical implementation and evaluation remain future steps, its design principles position SDA-FEF to support the next generation of intelligent, personalized, and interoperable healthcare solutions that contribute to improved health outcomes and quality of life.

## Data Availability

The original contributions presented in the study are included in the article/Supplementary Material, further inquiries can be directed to the corresponding author/s.
